# Biodegradation Kinetics of Tetrahydrofuran, Benzene, Toluene, and Ethylbenzene as Multi-substrate by *Pseudomonas oleovorans* DT4

**DOI:** 10.3390/ijerph120100371

**Published:** 2014-12-31

**Authors:** Dong-Zhi Chen, Yun-Feng Ding, Yu-Yang Zhou, Jie-Xu Ye, Jian-Meng Chen

**Affiliations:** 1 College of Biological and Environmental Engineering, Zhejiang University of Technology, Hangzhou 310032, China; E-Mails: dinghaoren1234@163.com (Y.-F.D.); yejiexu@zjut.edu.cn (J.-X.Y.); 2 School of Environmental Science and Engineering, Zhejiang Gongshang University, Hangzhou 310012, China; E-Mail: zyy0616@163.com

**Keywords:** biodegradation, tetrahydrofuran, benzene, toluene, ethylbenzene, kinetics model

## Abstract

The biodegradation kinetics of tetrahydrofuran, benzene (B), toluene (T), and ethylbenzene (E) were systematically investigated individually and as mixtures by a series of aerobic batch degradation experiments initiated by *Pseudomonas oleovorans* DT4. The Andrews model parameters, e.g., maximum specific growth rates (*μ*_max_), half saturation, and substrate inhibition constant, were obtained from single-substrate experiments. The interaction parameters in the sum kinetics model (SKIP) were obtained from the dual substrates. The *μ*_max_ value of 1.01 for tetrahydrofuran indicated that cell growth using tetrahydrofuran as carbon source was faster than the growth on B (*μ*_max_, B = 0.39) or T (*μ*_max_, T = 0.39). The interactions in the dual-substrate experiments, including genhancement, inhibition, and co-metabolism, in the mixtures of tetrahydrofuran with B or T or E were identified. The degradation of the four compounds existing simultaneously could be predicted by the combination of SKIP and co-metabolism models. This study is the first to quantify the interactions between tetrahydrofuran and BTE.

## 1. Introduction

Tetrahydrofuran is a polar ether that is widely used as a solvent for certain cements, pharmaceuticals, vinyl films, adhesives, vinylidene chloride polymers, and polyvinyl chlorides, as well as an intermediate in many industrial processes, specifically during the chemical synthesis of butyrolactone, 1,4-butanediol diacetate, motor fuels, pharmaceuticals, and insecticides [1]. This xenobiotic compound can cause health problems and even explosions due to its high water solubility and poor adsorption characteristics [2,3,4]. Tetrahydrofuran shows low to moderate acute toxicity potential in animals; however, inhalation of tetrahydrofuran vapors affects the central nervous system of human beings, which can result in headache, dizziness, and fatigue [5,6,7,8]. Yao *et al*. demonstrated that tetrahydrofuran exhibits adverse acute toxicity to microorganisms and has a significant negative impact on the performance of the activated sludge system even in a short time [9]. The National Toxicology Program claimed that some evidence of carcinogenic activity of tetrahydrofuran exist in male F344/N rats based on increased incidences of renal tubule adenoma or carcinoma after two-year inhalation [6]. Nevertheless, the controversy about tetrahydrofuran carcinogenicity still remains in governmental scientific committees internationally [5].

Biological process is becoming increasingly popular for the elimination of tetrahydrofuran from air and water environments because of its inherent green benefits and cost-effective potential. Bioscrubber is considered a suitable alternative in treating waste gas containing tetrahydrofuran because of the high water solubility of the substrate. However, the absorption solution is difficult to purify, which affects the efficiency of tetrahydrofuran removal. Tetrahydrofuran has been previously classified as “not readily biodegradable” for the cyclic structure and high bond energy of C–O (360 kJ/mol) [10]. Currently, its biodegradability has been illustrated as several strains were reported to have the ability to utilize tetrahydrofuranas the sole carbon source, including *Rhodococcus* sp. [11,12], *Pseudonocardiae* sp. [13,14,15], *Cordyceps sinensis* [16], and *Pseudomonas* sp. [17]. Among these tetrahydrofuran-degrading strains, *Pseudomonas oleovorans* DT4, which was recently isolated by Chen *et al.* [17], possessed the highest tetrahydrofuran-degrading activity in ever described strains, with doubling time of 2.7 h and maximum tetrahydrofuran degradation rate of 203.9 mg/(h·g dry weight). The microbial growth on the substrate mixture is a key subject of studies in the field of bioremediation and effluent treatment. However, limited studies have been conducted on the effects of co-contaminants on the degradation of tetrahydrofuran [18].

Benzene, toluene, and ethylbenzene, collectively known as BTE, are common toxic compounds emitted into the environment through spills and leakage from tanks and other releases [19]. The ubiquitous presence of these mono-aromatic compounds in chemical industrial processes as solvents and materials, causes the frequent co-existence of BTE and tetrahydrofuran. Since the last century, Jackson and Dwarakanath have validated this conjecture by reporting benzene and tetrahydrofuran as the most mobile of the six contaminants present at the Gloucester Landfill site [20]. In addition, a large number of industries generate waste gases containing both BTE and tetrahydrofuran in China based on our recent survey [20]. The need to understand the substrate interactions between BTE and tetrahydrofuran is very recent, and little research has been conducted on the potential effects between them. To the best of our knowledge, many different substrate interactions have been identified in the combinations of BTEX (“X” represents xylene) components that can alter the degradation rates through enhancement or competitive inhibition of substrate degradation in mixtures [21,22,23]. Bielefeldt and Stenselfirst focused their study on the quantitative evaluation of the biodegradation of a mixture of five BTEoXpX (ortho, para-xylene) substrates [24]. Deeb and Alvarez-Cohen conducted a study on the effect and interaction of the ethylbenzene on biodegradation of the benzene, toluene, and xylene isomers [25]. Little attention has been given to the effects of BTE on the kinetics of tetrahydrofuran biodegradation. However, their kinetic characteristics are beneficial for the proper design and improvement of a bioremediation process, so better understanding of the interactions and kinetics during the biodegradation of BTE and tetrahydrofuran is needed to enhance our risk assessment and remediation capabilities.

The biodegradation of more than one growth substrate by a pure strain was ambiguous; thus, the objective of this study was to develop kinetic models for the degradation of tetrahydrofuran and BTE under different substrate conditions. *P. oleovorans* DT4, which was isolated previously from a tetrahydrofuran-polluted soil and exhibited a great versatility in utilizing a variety of hydrocarbons, was selected as a model strain. Experiments were carried out where single-, two-, and four-component chemical mixtures with different ratios were used. The results of this study can be a useful reference in designing or optimizing cost effective and reliable bioreactors for the treatment of contaminated water and waste gas.

## 2. Experimental Section

### 2.1. Microbial Strain and Growth Medium

By virtue of its ability to utilize tetrahydrofuran as the sole carbon and energy source for growth [17], the recently isolated *P. oleovorans* DT4 that was deposited in the China Center for Type Culture Collection (M 209151) was used in this study.

The carbon-free mineral salts medium (MSM) used in this study was as described in the literature [17]. All chemicals used for media preparation were of high purity. Carbon source tetrahydrofuran was purchased from J&K Chemical, Ltd. (Shanghai, China). Benzene, toluene, and ethylbenzene were obtained from Sinopharm Chemical Reagent Co., Ltd. (Shanghai, China).

### 2.2. Kinetic Experiments

Kinetic experiments were conducted at 30 °C using 250 mL sealed glass serum vials containing 50 mL of MSM liquid with a self-sealing Teflon septum on the cap for sampling. A sufficient amount of headspace was provided to avoid oxygen-limiting conditions. If not specifically mentioned, the roughly similar levels of tetrahydrofuran and/or BTE were added to individual vials using high precision 5–10 μL syringes for each experimental run. Each bottle, with the exception of the control, was inoculated by 1 mL of bacterial culture at approximately the same time to obtain a final concentration of approximately 20.0 mg/L or 32.2 mg/L biomass in the liquid phase (50 mL) [22]. After inoculation, each bottle was placed in a temperature-controlled orbital shaker at 160 rpm and 30 °C. During the experiments, substrate consumption and cell growth were periodically monitored.

Control runs were conducted without microorganisms to discern BTE and tetrahydrofuran biodegradation from volatilization losses. Such losses were minor (5%), indicating that substrate removal was due to biodegradation. Dead controls with sterilized cells were also carried out, and the adsorption of the substrates onto the cells was neglected due to the low mass loss (less than 3%).

### 2.3. Analytical Methods

The change of B, T, or E concentration in the gas and liquid phases in each bottle can be related using Henry’s law as described by Hamed *et al.* [26]. The substrate that was sampled by a gas-tight syringe equipped with a side-sport needle was injected into a gas chromatograph (GC) (Agilent 6890) equipped with a silica HP-Innowax capillary column (30 m × 0.32 mm × 0.5 μm, J&W Scientific, USA) and a flame ionization detector. Nitrogen was used as the carrier gas at a flow rate of 1 mL/min. Oven temperature was controlled at a constant of 90 °C, whereas the injector and detector temperatures were set at 200 °C and 250 °C, respectively. For the determination of BTE in a mixture, the substrate in the vial was converted from its concentration in the headspace by partition coefficients, which were previously calibrated using an established calibration curve about one substrate mixing with different total concentrations of the other co-contaminants. Tetrahydrofuran is highly polar and is freely miscible with water, so the supernatant of the culture after centrifugation was directly injected into the GC for tetrahydrofuran determination [17].

Biomass concentrations in liquor (expressed in mg dry weight/L) were measured by optical density (OD) at 600 nm using an UV spectrometer (HITACHI U-2910 Double Beam UV/Vis spectrophotometer, Tokyo, Japan). The OD measurements were then converted to dry weight concentrations using an established calibration curve.

### 2.4. Kinetic Models and Parameter Estimation

The kinetic model parameters are determined by many factors, such as inoculum size, substrate concentration, culture history, and cell cultivation (e.g., batch, continuous). Considering that the continuous cultures failed to estimate the maximum specific growth rate, we chose to use batch cultures to measure biodegradation kinetics, evaluate models, and determine model parameters. For batch degradation, the classical Monod model (Equation (1)) was applied to study the biodegradation kinetics of single-substrate *i*.

(1) $$\mu_{i}=\frac{\mu_{\text{max }i}S_{i}}{K_{Si}+S_{i}}$$

where *μ*_max_ is the maximum specific growth rate, *K_S_* is the saturation coefficient, and *S* is the substrate concentration. Single-substrate degradation experiments can be used to estimate the kinetic parameters *μ*_max_ and *K_S_* from the experimental specific growth rates (*μ*) and substrate concentrations (*S*) for each substrate.

Although Monod model is convenient, its suitability for fitting the kinetic parameters *μ*_max_ and *K_S_* is based on the assumptions that only substrate concentration is the rate limiting factor utilized and that the alterations of the culture behavior are caused by the variation in substrate concentration [27]. However, considering the toxic nature of tetrahydrofuran and BTE and the possibility of substrate inhibition in this study, Andrews model (Equation (2)), which is a modified Monod model, may provide a better fit to the experimental data obtained from the single-substrate experiments [28].

(2) $$\mu_{i}=\frac{\mu_{\text{max }i}S_{i}}{K_{Si}+S_{i}+S_{i}^{2}/K_{i}}$$

where *K_I_* is the inhibition coefficient. If *K_I_* >> *S_i_*, the Andrews model is transferred to Monod one.

For the substrate mixtures, the interaction parameters were determined using the sum kinetics with interaction parameter (SKIP) model in Equation (3) [22].

(3) $$\mu_{i}=\frac{\mu_{\text{max }i}S_{i}}{K_{Si}+S_{i}+I_{i,j}S_{I}}$$

Compared with the competitive and non-competitive inhibition models, the mathematical description gives an adjustment of the non-specific interaction between substrates by incorporating the interaction parameter *I_i,j_* (estimated from Equation (3)), which indicates the degree to which substrate *i* affects the biodegradation of substrate *j*, with the large values referring to stronger inhibition [21]. Additionally, experimentally obtained specific growth rates can be plotted as a function of substrate concentrations and fitted to Equation (2) and Equation (3) to estimate the kinetics parameters *μ_max_*, *K_S_*, and *K_I_*. The three parameters in Equation (2) and Equation (3) were the same in the single-substrate experiments. Depletion rate (*v*) of growth substrates for a given substrate *i* can be calculated from the available experimental parameters *μ_i_*, biomass concentration (*X*), and cell yield (*Y_X/Si_*) using Equation (4).

(4) $$v=\frac{dS_{i}}{dt}=-\frac{\mu_{i}X}{Y_{X/S_{i}}}$$

In the case of co-metabolism, the specific growth rate of a single non-growth substrate is well known to be zero because the substrate is not metabolized for energy purpose. Therefore, a special expression (Equation (5)) is selected to describe the disappearance of a co-metabolized substrate by incorporating an estimated parameter *T_g_^C^*, which is called growth substrate transformation capacity (mg_N_/mg_G_) [22].

(5) $$\frac{dS_{N}}{dt}=-(T_{g}^{C}(\frac{dS_{G}}{dt}(\frac{1}{X}))(\frac{S_{N}}{K_{S_{N}}+S_{N}})X$$

where *S_N_* and *S_G_* are the concentration of non-growth substrate and that of growth substrate, respectively.

All the mathematical kinetic coefficients were obtained from nonlinear regression analysis by OriginLab 8.0. The adequacy of the kinetic parameter estimated from the model was determined by viewing the residual versus predicted plot mean square regression ratio tests, *R^2^* values, and parameter significance to the 95% interval.

## 3. Results and Discussion

### 3.1. Determination of the Kinetics Model for Single Substrate

As described previously, B and T could be utilized as growth substrates by *P. oleovorans* DT4, whereas co-metabolism of E occurs with tetrahydrofuran addition [18]. Therefore, kinetic experiment of single substrate was conducted by using tetrahydrofuran, B, or T as the sole carbon resource in DT4.

**Figure 1:**
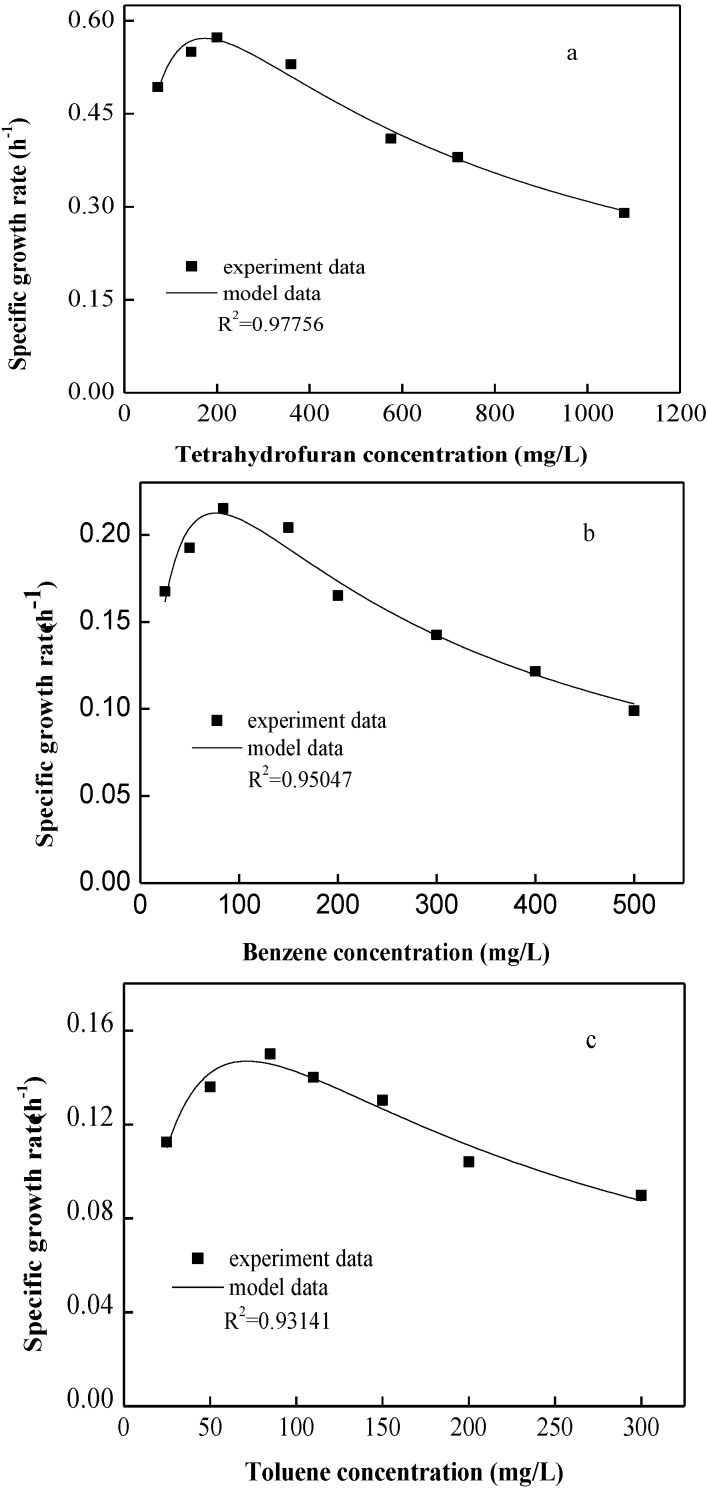
Specific degradation rate of strain DT4 at various concentrations of (**a**) tetrahydrofuran, (**b**) B, and (**c**) T. The initial culture density in each bottle was 20 mg/L.

Figure 1 presents the substrate degradation profiles as a function of different initial concentrations for single-substrate experiments. The *R*^2^ values for tetrahydrofuran, B, and T were 0.98, 0.95, and 0.93, respectively, indicating good correlation between the experimental and predicted values obtained from the Andrews model. Therefore, the kinetic model describes the experimental data accurately, which is further confirmed by the residual analysis with no significant trend.

Table 1 shows a summary of the Andrews kinetic parameters. To the best of our knowledge, our group is the first to report on the kinetics of tetrahydrofuran biodegradation. Hence, the comparison of kinetic parameters was only conducted for B and T. The maximum specific growth rates, *μ*_max_, obtained from the different references for B and T were in the range of 0.3–0.7 and 0.4–0.9 h^−1^; similar values of B (0.39) and T (0.33) in this study fall within these ranges. Compared with previous studies, *P. oleovorans* DT4 has relatively high activity of degrading B with *μ*_max_ of 0.39 h^−1^, which is nearly the same as that reported by Littlejohns and Daugulis under similar conditions [22]. However, *μ*_max_ of tetrahydrofuran was higher (1.01 h^−1^) than that of B and T, implying that tetrahydrofuran was the preferred carbon source by DT4 among the three compounds. Most of the reported values of *μ*_max_ for the same substrate biodegradation were quite different, showing a wide variation range, which was possibly caused by applying different experimental conditions (type of microorganism or concentration of substrate). The inhibition coefficients *K_I_* for tetrahydrofuran, B, and T were calculated to be 455.1, 184.0, and 113.6 mg/L, respectively, which indicated that the inhibition effect might be observed only in a high concentration range [21]. The relatively high *K_I_* for tetrahydrofuran indicated that the culture DT4 was less sensitive to substrate inhibition [29]. The values of *K_S_* for B and T degradation by *P. oleovorans* DT4 were 31.83 and 44.83 mg/L, respectively, compared with other described strains that had values ranging from 0.12–27.57 mg/L and 3.98–34.12 mg/L, respectively, indicating that DT4 could ultilizesubstrates with higher levels.

**Table 1:** Biodegradation model parameter values for tetrahydrofuran, B, and T by various microorganisms as described in the literature.

Substrate	*μ*_max_ (h^−1^)	*K_S_* (mg/L)	*K_I_* (mg/L)	Microorganism	Reference
Tetrahydrofuran	1.01	65.95	455.1	*P. oleovorans* DT4	this study
B	0.39	31.83	184.0	*P. oleovorans* DT4	this study
	0.335	3.17	--	*P. fragi* B1	Chang *et al.* [30]
	0.44	3.36	--	*P. putida* O1	Oh *et al.* [21]
	0.73	0.12	--	*P. putida* F1	Reardon *et al.* [31]
	0.62	1.65	180	*P. putida* F1	Abuhamed *et al.* [21]
	0.44	27.57	--	mixed bacteria	Littlejohns and Daugulis [22]
T	0.33	44.83	113.6	*P. oleovorans* DT4	this study
	0.437	6	1980	*P. putida* ATCC23973	Choi *et al.* [32]
	0.42	3.98	42.8	*P. putida* 54G	Mirpuri and Bryers [33]
	0.86	13.8	--	*P. putida* F1	Reardon *et al*. [31]
	0.61	6.47	88	*P. putida* F1	Abuhamed *et al.* [21]
	0.60	34.12	--	mixed bacteria	Littlejohns and Daugulis [22]

### 3.2. Degradation Kinetic of Tetrahydrofuran with Mixture of Benzene, Toluene, or Ethylbenzene

**Figure 2:**
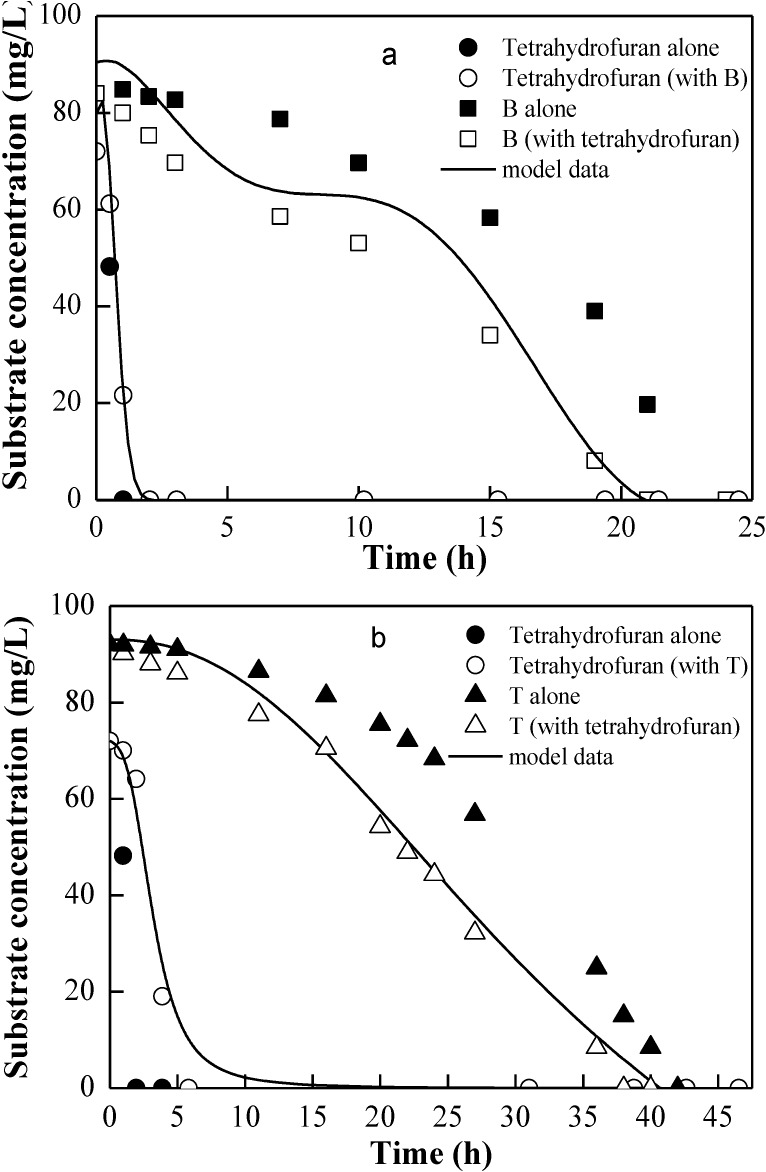
Dual degradation of tetrahydrofuran with mixture of (**a**) B or (**b**) T presented by experimental data (shapes) and sum kinetics with interaction parameter (SKIP) model (lines). The initial culture density in each bottle was 20 mg/L.

**Figure 3:**
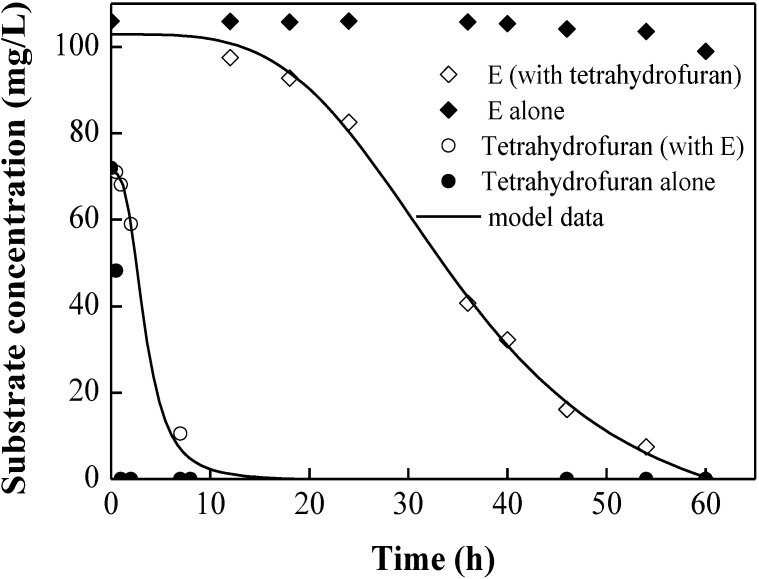
Dual degradation of tetrahydrofuran and E presented by experimental data (shapes) and sum kinetics with interaction parameter (SKIP) (lines). The initial culture density in each bottle was 20 mg/L.

Trigueros *et al.* reported that the SKIP model represents experimental data better than other models in the determination of kinetic parameters for BTE, with its main advantage being the consideration of substrate interaction [34]. This model was also applied to represent the effect of BTE on tetrahydrofuran degradation in this work. Figure 2 and Figure 3 illustrate the dual-substrate (tetrahydrofuran with B, T, or E at roughly similar levels) experimental results and the predicted profiles of SKIP model. The SKIP model provides a good fit for the dual utilization (tetrahydrofuran and B or tetrahydrofuran and T).

As shown in Figure 2a,b, tetrahydrofuran was consumed before BT degradation. Tetrahydrofuran was depleted first with similarity to the case of diauxic growth. As a preferred substrate, tetrahydrofuran enhanced the consumption of other substrates in this work. Meanwhile, dual-substrate experiments containing E, which could not be utilized as a sole carbon source by DT4 (Figure 3), showed that E was co-metabolized in the presence of tetrahydrofuran. Therefore, Equation (5) was used to describe the disappearance of E in the mixture with tetrahydrofuran. The experimental substrate degradation data were fitted accurately by co-metabolism and SKIP models (Figure 3). Compared with single-substrate experiments, the simultaneous degradation of tetrahydrofuran and BTE resulted in an inhibitory effect of BTE on tetrahydrofuran degradation, as shown by the interaction parameter *I_i,j_* in Table 2. A larger value of the interaction parameter *I_i,j_* shows a higher degree of inhibition. *I_B,THF_*, *I_T,THF_*, and *I_E,THF_* interactive parameter values are 35.4, 48.9, and 65.3, respectively, indicating that tetrahydrofuran degradation was affected by B,T, and E in ascending order of inhibitory effects. These results were slightly similar to the reports by Trigueros *et al.* [34]. Bielefeldt and Stensel also reported inhibition interaction effects during BTE and *o*-xylene biodegradation [24]. The experimental data were fitted through a competitive inhibition model proposed by Yoon *et al.* [35]. The models that account for competitive inhibition, non-competitive inhibition, and un-competitive inhibition among dual substrates were determined, and no model could accurately fit the experimental data for the dual-substrate biodegradation (data not shown). In the SKIP model for dual substrates, *I_E,B_* and *I_E,T_* were respectively estimated as 10 and 4.5, deducing that E might be the strongest inhibitor between the BTE biodegradable compounds [34]. Conversely, tetrahydrofuran had a relatively slight effect on the biodegradation of BTE, with smaller values of *I_THF,B_* = −0.38, *I_THF,T_* = −0.23 and *I_THF,E_* = −1.5. Such values indicated that tetrahydrofuran had an enhancing effect on BTE biodegradation.

Thus, the SKIP model was proven to be a suitable and accurate model type that could be used to describe the interactions of dual-substrate degradation. Equation (5) was suitable to describe the co-metabolism of E in the presence of tetrahydrofuran. The kinetic parameters and substrate interactions determined from single and dual-substrate experiments were validated by combining them to model the degradation of BTE and tetrahydrofuran simultaneously.

**Table 2:** Interaction parameters estimated from sum kinetics with interaction parameter (SKIP) model for dual substrates.

Compounds	*I_i,j_*	Microorganism	Reference
Tetrahydrofuran and BTE	*I_THF,B_*^2^ = −0.38, *I_B,THF_* = 35.4	*P. oleovorans* DT4	this study
	*I_THF,T_* = −0.23, *I_T,THF_* = 48.9		
	*I_THF,E_* =−1.5, *I_E,THF_* = 65.3		
BT and Phenol (P)	*I_T,P_* = 55, *I_P,T_* = 0.01	*P. putida* F1	Reardon *et al.* [31]
	*I_T,B_* = 5, *I_B,T_* = 0.01		
	*I_B,P_* = 18.5, *I_P,B_* =0.01		
BTP	*I_T,P_* = 0.14, *I_P,T_* = 1.03	*P. putida* F1	Abuhamed *et al.* [21]
	*I_T,B_* = 5.16, *I_B,T_* = 0.49		
	*I_B,P_* = 0.27, *I_P,B_* = 1.08		
BTE	*I_T,B_* = 2	mixed bacterial	Littlejohns and Daugulis [22]
	*I_B,T_* = −0.4		
	*I_E,B_* = 4		
Phenol (P), Vanillin (V), Oxalic (O) and Formic acid (F) ^1^	*I_P,V_* = 0.03	*P. putida* CECT324	Martin *et al.* [36]
	*I_V,O_* = 10^5^		
BTE	*I_T,B_* = 1, *I_B,T_* = 0.0023	*P. putida* F1	Trigueros *et al.* [34]
	*I_E,B_* = 10, *I_B,E_* = 0.175		
	*I_T,E_* = 0.025, *I_E,T_* = 4.5		

^1^ Interaction parameters were chosen at the condition of pH 5 and 25°C; ^2^ THF here and those mentioned in the following paragraphs represent tetrahydrofuran.

### 3.3. Degradation Kinetic of the Quaternary Substrate Experiment

**Figure 4:**
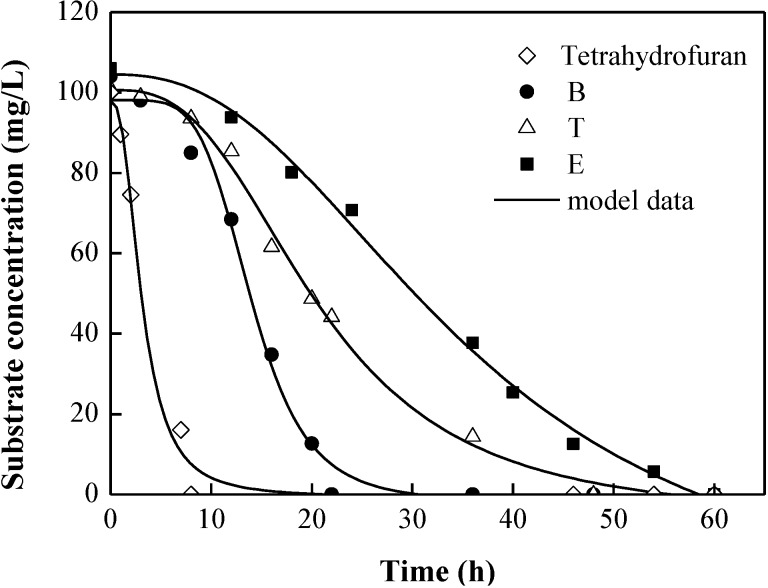
The simultaneous degradation of tetrahydrofuran, benzene (B), toluene (T), and ethylbenzene (E) in quaternary substrate experiment (shapes) and model (lines). The initial culture density in each bottle was 32.2 mg/L.

The SKIP model proposed to describe the interaction of dual substrate has been extended to cover that of more substrates. The model was applied by Reardon *et al.* [31] and Abuhamed *et al.* [21] to explore the biodegradation kinetics of B, T, and phenol as mixed substrates with *P. putida*. It was also used by Martín *et al.* for modeling the growth of *P. putida* in mixtures of formic acid, vanillin, phenol, and oxalic acid [36]. Model parameters determined from single- and dual-substrate experiments were sufficient to predict accurately the outcome of more substrate mixtures using the SKIP model. Figure 4 shows the experimental data and corresponding SKIP/co-metabolism model predictions for the simultaneous degradation of all four substrate mixtures by *P. oleovorans* DT4. The total concentration of the contaminants in the quaternary substrate experiment was higher than that in the dual-substrate one. Thus, the initial culture biomass increased to make the initial substrate-to-biomass ratio sufficiently high to obtain the intrinsic and unique parameter estimates of kinetics. The kinetics parameters obtained from the dual-substrate degradation provide an adequate prediction of the experimental data for the degradation of all four compounds (BTE and tetrahydrofuran). Compared with single-substrate biodegradation experiment, they prove the hypothesis that BTE and tetrahydrofuran compound interactions occur in such complex system.

As shown in Figure 4, the model provides a good fit with a relative high *R*^2^ value of more than 0.94, and it matches most sets of the measured data well. Among the four substrates, tetrahydrofuran was still the first to be consumed and disappear, followed by B, T, and E. The maximum biodegradation rate for tetrahydrofuran greatly decreased and was almost one-third of that for tetrahydrofuran in single-substrate biodegradation. This result implied a relative challenge for *in situ* bioremediation processes and for the treatment of industrial waste gas because co-contamination with other pollutants was frequently found in tetrahydrofuran-contaminated sites. The biodegradation of E was slightly more enhanced by the presence of other aromatic compounds (BT) than by that of tetrahydrofuran (Figure 3), possibly due to the induction of required catabolic enzymes by the homologous compound. The similar result has been reported by Littlejohns and Daugulis [22]. The rates of BT degradation slightly decreased during the quaternary-substrate experiment compared with the dual-degradation experiments shown in Figure 2, suggesting that the degree of inhibition of E on BT biodegradation was stronger than the degree of enhancement of tetrahydrofuran on BT. Consequently, the parameter *I_i,j_* allows the quantification of the interactions, including the inhibition and enhancement between BTE and tetrahydrofuran, which was considered as the main advantage of the SKIP model.

## 4. Conclusions

The co-contamination of BTE in tetrahydrofuran-contaminated sites (groundwater and waste gas) has recently become a well-known problem. This study is the first to investigate the kinetic characteristics of interactions between tetrahydrofuran and BTE during their aerobic biodegradations. The direct metabolism of tetrahydrofuran, B, and T as the single substrate followed the Andrews model, which included inhibition terms. Several interactions, such as enhancement, inhibition, and co-metabolism in dual systems containing tetrahydrofuran and B/T/E, were identified by comparison with single-substrate degradation. The interactions between tetrahydrofuran and B (or T) in dual-substrate mixtures could be described by the SKIP model with the incorporation of an interaction parameter. The co-metabolism of E was modeled mathematically by introducing substrate transformation capacity. The kinetic parameters obtained from single- and dual-substrate mixture experiments fitted the four-substrate mixture experimental data well.
